# Environmental exposure assessment in European birth cohorts: results from the ENRIECO project

**DOI:** 10.1186/1476-069X-12-8

**Published:** 2013-01-23

**Authors:** Ulrike Gehring, Maribel Casas, Bert Brunekreef, Anna Bergström, Jens Peter Bonde, Jérémie Botton, Cecile Chévrier, Sylvaine Cordier, Joachim Heinrich, Cynthia Hohmann, Thomas Keil, Jordi Sunyer, Christina G Tischer, Gunnar Toft, Magnus Wickman, Martine Vrijheid, Mark Nieuwenhuijsen

**Affiliations:** 1 Institute for Risk Assessment Sciences, Utrecht University, Utrecht, The Netherlands; 2 Centre for Research in Environmental Epidemiology (CREAL), Barcelona, Spain; 3 Hospital del Mar Research Institute (IMIM), Barcelona, Spain; 4 Spanish Consortium for Research on Epidemiology and Public Health (CIBERESP), Pamplona, Spain; 5 Julius Center for Health Sciences and Primary Care, University Medical Center Utrecht, Utrecht, The Netherlands; 6 Institute of Environmental Medicine, Karolinska Institutet, Stockholm, Sweden; 7 Department of Occupational and Environmental Medicine, Bispebjerg University Hospital, Copenhagen NV, Denmark; 8 INSERM U1085 - IRSET, University of Rennes I, Rennes, France; 9 Helmholtz Zentrum, München & German Research Centre for Environmental Health, Institute of Epidemiology I, Neuherberg, Germany; 10 Institute of Social Medicine, Epidemiology and Health Economics, Charité University Medical Center Berlin, Berlin, Germany; 11 Universitat Pompeu Fabra (UPF), Barcelona, Spain; 12 Department of Occupational Medicine, Aarhus University Hospital, Aarhus, Denmark

**Keywords:** Environment, Europe, Exposure assessment, Birth cohort, Review

## Abstract

Environmental exposures during pregnancy and early life may have adverse health effects. Single birth cohort studies often lack statistical power to tease out such effects reliably. To improve the use of existing data and to facilitate collaboration among these studies, an inventory of the environmental exposure and health data in these studies was made as part of the ENRIECO (Environmental Health Risks in European Birth Cohorts) project. The focus with regard to exposure was on outdoor air pollution, water contamination, allergens and biological organisms, metals, pesticides, smoking and second hand tobacco smoke (SHS), persistent organic pollutants (POPs), noise, radiation, and occupational exposures. The review lists methods and data on environmental exposures in 37 European birth cohort studies. Most data is currently available for smoking and SHS (N=37 cohorts), occupational exposures (N=33), outdoor air pollution, and allergens and microbial agents (N=27). Exposure modeling is increasingly used for long-term air pollution exposure assessment; biomonitoring is used for assessment of exposure to metals, POPs and other chemicals; and environmental monitoring for house dust mite exposure assessment. Collaborative analyses with data from several birth cohorts have already been performed successfully for outdoor air pollution, water contamination, allergens, biological contaminants, molds, POPs and SHS. Key success factors for collaborative analyses are common definitions of main exposure and health variables. Our review emphasizes that such common definitions need ideally be arrived at in the study design phase. However, careful comparison of methods used in existing studies also offers excellent opportunities for collaborative analyses. Investigators can use this review to evaluate the potential for future collaborative analyses with respect to data availability and methods used in the different cohorts and to identify potential partners for a specific research question.

## Introduction

Pregnancy and birth cohort studies provide the possibility of repeated measurements of health outcomes and exposures at different time points from pregnancy through childhood and adolescence into adulthood. Therefore, they present an ideal framework for the prospective study of the effects of environmental exposures on the health and development of children. Furthermore, they allow the study of temporal variability in exposure. If temporal variability is sufficient, the relevance of exposure at different time points (e.g. prenatal and postnatal) and the health impact associated with changes in exposure can be investigated, which may help to focus preventive actions.

Currently, many European birth cohort studies of children’s environment-health relationships exist and provide evidence for relationships that can be used to develop strategies to reduce environmental exposures and to improve health. However, single studies often lack statistical power to lead to conclusive results on their own, in particular when health outcomes and/or exposures with low prevalence are studied. Combining data from different studies is a powerful remedy for this. Also, collaboration between birth cohorts enables us to replicate and corroborate or refute findings as well as to explore reasons for heterogeneity. Therefore, there is an urgent need to evaluate, and where possible combine, the existing data, methods and tools from European birth cohort studies in order to evaluate possible links between environmental exposures and health [1].

The assessment of exposure is a crucial element of the study of the potential adverse effects associated with exposure. Error in exposure measurements reduces the statistical power of a study [2] and the estimated effect is likely to be smaller than the true effect [3], both increasing the likelihood that real associations are not detected. Exposure can be assessed by means of direct approaches including biological and personal monitoring, by means of indirect approaches including environmental monitoring and modeling as well as the use of questionnaires and diaries or a combination thereof [4]. Exposure assessment by means of (repeated) individual environmental or biological monitoring, however, is costly and therefore usually not feasible in all participants of large cohort studies. This means that making informed decisions of what to measure in how many study subjects is crucial.

As part of the European Union funded Environmental Health Risks in European Birth Cohorts (ENRIECO) project, an inventory of the environmental exposure and health data in existing European birth cohort studies was made [5]. The objective of the present review is to analyze the environmental exposure assessment in the birth cohort studies in detail. The review covers established exposures (e.g. ambient air pollution, environmental tobacco smoke, allergens and biocontaminants, metals, persistent organic pollutants (POPs) and noise) as well as exposures that more recently became the focus of epidemiological research (e.g. electromagnetic fields, phthalates and phenols). The emphasis is on comparison of methods currently used in birth cohort studies leading to recommendations for improvement. We focus on methodological issues such as comparability, validity, (dis-)agreement between methods, ability to predict concentrations in earlier or later periods, and timing of exposure assessment that are relevant to several exposures rather than exposure-specific issues.

## Methods

### Participating cohorts and inventory

A detailed description of the cohorts included in the ENRIECO project has been published elsewhere [5]. In brief, cohorts were included in an inventory if they a) collected data on at least one of the following exposures: air pollution, water contamination, allergens and microbial agents, metals, pesticides, POPs, other chemical pollutants, noise, and radiations; b) started enrolment of mothers into the cohort during pregnancy or at birth; c) included in their protocol at least one follow-up point after birth with direct contact with mothers and children; d) included at least 200 mother-child pairs; and d) were based in a European country. Between March 1, 2009 and February 15, 2011 37 different European birth cohorts (counting the cohorts of the Faroes, the old and the new INMA cohorts as one cohort each) completed inventory questionnaires with detailed information on the study protocol, exposure and health outcome assessment [5]. A web-based searchable inventory database is now publically available on http://www.birthcohortsenrieco.net. The information provided by the inventory has been approved by the cohorts.

### Evaluation of exposure information

The evaluation was divided in 11 exposure groups: outdoor air pollution, water contamination, allergens and microbial agents, metals, pesticides (including persistent pesticides), POPs, other chemical pollutants (e.g. phthalates and phenols), smoking and second hand tobacco smoke (SHS), noise, radiation and occupational exposures. The evaluations were conducted by experts from several fields relevant to the topic of this review, including environmental and occupational epidemiology, toxicology, public health, medical sciences (more specifically, pediatrics), sociology, psychology, and biostatistics. A full list of the names of the experts who participated in the different working groups is presented in Additional file 1: Table S1.

The aims of the evaluations were a) to describe the environmental exposure assessment in European birth cohort studies including methods used, timing and coverage (number of cohorts and number of subjects within cohorts with exposure information) of exposure assessment; b) to discuss the exposure assessments used in the birth cohort studies in terms of agreement between direct and indirect methods, timing and temporal variability of exposure measurements, exposure at non-residential addresses and time-activity patterns, c) to discuss the potential for collaborative analyses within the birth cohort studies including examples of successful collaborations; and d) to discuss areas of interest for future analyses including new data collection and new methods.

## Results

### Exposure assessment methods currently used in European birth cohorts

A description of the basic characteristics of the cohorts has been presented elsewhere [5]. The exposure assessment methods used in the cohorts are summarized in Table 1. Exposure modeling is becoming the method of choice for air pollution exposure assessment. The focus currently is on nitrogen oxides and fine particular matter (Additional file 1: Table S2). Exposure to cat and dog allergens, molds, radiation, smoking and SHS, noise, and occupation of the parent(s) were mainly assessed by means of questionnaires, whereas exposure to house dust mites was exclusively assessed by means of environmental monitoring. Information on environmental monitoring for assessment of allergen and microbial exposures is provided in Additional file 1: Table S3. Sometimes, biomonitoring and/or environmental monitoring were performed in addition to questionnaires to assess SHS exposure (Additional file 1: Table S4). Exposure to metals, POPs and other chemicals such as phthalates and phenols was mainly assessed by means of biomonitoring. More detailed information has been published elsewhere [5-7]. Exposure to water contaminants was mostly assessed by means of a combination of environmental measurements and questionnaire information on water use. Disinfection by-products were studied most (Additional file 1: Table S5). Because exposure to pesticides occurs through several pathways (household use, food, occupational exposure, residential exposure through agricultural activities, etc.) the assessment methods used in the cohorts are multiple and variable.

**Table 1:** Description of exposure assessment in the birth cohorts by exposure topic

**Exposure topic**	N *	**Description**
Outdoor air pollution	27	·Many cohorts assessed outdoor air pollution exposure (27 cohorts).
		·Air pollution modeling is becoming increasingly the method of choice: land-use regression modeling (18 cohorts) and dispersion modeling (10 cohorts).
		·Sixteen cohorts are currently participating in the collaborative EU-funded ESCAPE project that adds land-use regression modeling of nitrogen oxides, particulate matter, soot and particle composition to existing cohort studies using a standardized protocol.
		·Most cohorts currently have data on exposure during pregnancy and/or early life.
Water contamination	13	·Disinfection by-products were studied most.
		·Exposure assessment usually by means of a combination of questionnaires *and* individual measurements or routinely collected measurement data (8 cohorts).
		·Validation by means of biomonitoring in a small number of subjects (3 cohorts).
		·Most studies assessed exposure during pregnancy.
Allergens & microbial agents	27	·Exposure to cat and dog allergen was assessed by means of questionnaires in all cohorts and by means of measurements in house dust samples in 9 and 4 cohorts, respectively.
		·Mite allergen levels were measured in settled house dust samples in 10 cohorts.
		·Mold exposure was mainly assessed by means of questionnaires.
		·Exposure was assessed during infancy and/or early childhood in most studies.
Metals	20	·Most cohorts have analyzed the effects of low-level environmental exposure to mercury (Hg; 15 cohorts) and lead (Pb; 16 cohorts); little attention to other metals.
		·Exposure was mainly assessed by means of biomonitoring. Five cohorts used questionnaires, two of them in addition to biomonitoring,
		·There are well-standardized protocols for most of the metals.
		·Inductively coupled plasma mass spectrometry (ICP-MS) and atomic absorption spectroscopy (AAS) were used most.
		·Most measurements were performed in cord blood; other non-invasive matrices such as hair and urine are gaining attention.
Pesticides ^a^	18	·Many studies assessed household use (16 cohorts); and occupational exposure (13 cohorts), fewer cohorts assessed dietary exposure (6 cohorts) or residence proximity to crops (2 cohorts).
		·Exposures via household use, occupational exposure and diet, were mainly assessed by means of questionnaires.
Persistent organic pollutants	19	·Exposure assessment by means of high performance liquid chromatography (HPLC) measurements in biological samples with adjustment for lipid content.
		·Variation between studies with regard to sampling medium, timing of sample collection and lipid adjustment.
		Most data available for polychlorinated biphenyl (PCB) and dichlorodiphenyltrichloroethane (DDT).
Other chemical exposures ^b^	17	·Few cohorts have measured these contaminants (13 cohorts), but this is a rapidly developing field and more cohorts are planning to assess exposure to other chemicals (7 cohorts).
		·Exposure was mainly assessed by means of biomonitoring.
		There is heterogeneity with regard to the type of biological media used and the timing of the exposure measurement.
Radiation	12	*Ionizing radiation*
		·Mainly assessed by questionnaire: maternal occupational exposures (3 cohorts), prenatal medical ionizing radiation exposures (6 cohorts); 2 cohorts currently plan to ask questions about medical radiation exposures in children.
		·1 cohort is planning to assess residential radon exposure using geographical methods.
		·No standardized questionnaires or protocols in this field.
		*Ultraviolet (UV)*
		·Only six cohorts are collecting UV-related data through questionnaire questions on sunburn in children, use of sun beds during pregnancy, and time spent outdoors.
		·None of the cohorts collect data on maternal and child skin type, sunscreen use, or clothing.
		·Standard questionnaires are not available.
		*Non-ionizing radiation*
		·Very few cohorts assess exposure to non-ionizing radiation: 2 cohorts include occupational electromagnetic field (EMF) exposure in their questionnaires, 2 cohorts assess extreme low frequency (ELF) exposure to overhead high-voltage power lines through geographical information from electricity companies, 2 cohorts include questions about mobile phone use of the mother during pregnancy and 4 on children’s mobile phone use.
		·A few cohorts have started using base-station maps combined with information from home appliances and personal radio frequency (RF) exposimeters, in order to estimate whole body RF/ELF-EMF exposure.
		·There are no standardized or validated questionnaires, models or protocols in use at this moment.
Smoking and second hand tobacco smoke	37	·All cohorts have information about exposure during pregnancy and 29 cohorts in addition assessed exposure at different periods during infancy and childhood.
		Assessment mainly by questionnaire; cotinine measurements in biological samples (mainly urine) in 14 cohorts.
Noise	14	·All cohorts used questionnaire assessments, mainly about noise annoyance.
		·5 cohorts used noise propagation modeling or noise maps.
		·Traffic is the source of noise that has been studied most.
		·Most cohorts assessed exposure during pregnancy.
Occupational exposures	33	·All cohorts have information on maternal occupation and most cohorts (n=26) have information on paternal occupation for at least one point in time.
		·Data mainly collected by means of questionnaires (most often job title; sometimes checklist occupation or occupational exposures).
		·Coding of maternal job title (n=17) or use of Job Exposure Matrices (JEM) (n=8) planned/done in a number of studies.

* N = Number of cohorts with exposure assessment, counting the cohorts of the Faroes, the old INMA cohorts and the new INMA cohorts as one cohort each; ^a^ Organochlorine pesticides are under persistent organic pollutants; ^b^ brominated flame retardants, perfluorinated compounds phthalates and phenols.

### Coverage of different exposure assessment methods

Exposure data was generally available for the vast majority of the study participants if exposure was assessed by means of exposure modeling, questionnaires, routinely collected data or a combination thereof (see e.g. http://www.enrieco.org). Biomonitoring was sometimes performed in addition to other methods (e.g. for assessment of exposure to water contaminants, smoking and SHS exposure) usually in small subsets of the study population for validation purposes (Additional file 1: Tables S4 and S6). Also for other exposures such as metals, POPs, phthalates and phenols, where exposure assessment largely relied on biomonitoring, biomonitoring was often restricted to subsets of the cohorts due to the costs involved (Additional file 1: Table S6). Nevertheless, performing biomonitoring (and other exposure assessments that require large resources) in subsets of prospective cohort studies can still be very efficient, if for example the outcome of interest is rare and the subset has been selected according to a nested case–control design, which is superior to a conventional case–control design as it is less prone to recall and selection bias [8]. Moreover, the internal human dose can be estimated more efficiently and precisely by biomonitoring as compared to chemical analyses of different environmental matrices at different time points if exposure occurs through multiple pathways.

### Comparison of direct and indirect methods

Questionnaires for the assessment of pet allergen and mold exposure as well as SHS exposure have been compared with environmental monitoring (e.g. house dust and air sampling) in a number of cohort studies that are part of ENRIECO, but also in other studies. Despite some misclassification, questionnaire reports were found to be an inexpensive and valid estimate of residential SHS exposure among preschool and school children [9-11]. Questionnaires were also found to be a valid method of assessing exposure to drinking water in pregnant women [12]. In contrast, questionnaire-reported cat and dog ownership is a relatively poor measure of pet allergen levels in house dust [13-16]. Similarly, it has been shown that questionnaire data cannot be used as a surrogate for measurements of specific microbial agents such as endotoxin, gram positive bacteria and mold components in house dust [17-19].

### Timing of exposure assessment and time-activity patterns

Most exposures were mainly assessed during pregnancy and/or at birth. Exposure to outdoor air pollution has mainly been assessed for pregnancy and/or early life; exposure to allergens and biological contaminants was mainly assessed during infancy and early childhood (Additional file 1: Tables S2 and S3).

At present, repeated exposure assessments are available for a limited number of exposure topics (e.g. outdoor air pollution, allergens and microbial agents, smoking and SHS exposure, and phenols). Time-activity patterns, exposure at non-residential addresses and residential mobility are currently rarely included in the assessment.

### Potential for collaborative analyses

Combined analyses have been successfully performed/are being performed within the framework of EU-collaborative projects such as GA2LEN (allergens [20]), TRAPCA (outdoor air pollution [21]), ESCAPE (outdoor air pollution [22,23]), HIWATE (water contamination [24]) AIRALLERG (allergens, biological contaminants and indoor air pollution [25,26]), and HITEA (indoor biological agents, http://www.hitea.eu/). In addition, for exposure to molds [27] second hand tobacco smoke [28], and POPs [6], combined analyses have been performed as part of case studies within the current ENRIECO project to explore the feasibility, potentials and difficulties of merging partly heterogeneous data from different European pregnancy and birth cohort studies. For ionizing, non-ionizing and UV radiation, as well as other chemical contaminants there is currently not sufficient data available in the cohorts for data pooling, but many measurements are ongoing and comparison studies may be feasible within the next few years.

### Recommendations for future research

Recommendations for future research are presented in Table 2. The performance of validation studies, the assessment of the role of the timing of the exposure, and the inclusion of time-activity pattern and non-residential exposures in the exposure assessment have the highest priority.

**Table 2:** Recommendations for methods, evaluations of collaboration with existing data, and areas of interest for future work including new data/new methods by exposure topic

**Exposure topic**	**Recommendations for methods**	**Collaboration with existing data**	**Areas of interest for future work**
Outdoor air pollution	·Exposure modeling is currently the state-of the art method	·Within the ESCAPE project, a standardized exposure assessment is being added to a number of birth cohort studies and will soon be linked to existing health data in these cohorts; pooled analyses will be performed for a number of health endpoints	·Assessment of long-term exposure to ultrafine particles, which are currently not being assessed within cohort studies.
	·Few studies so far compared land-use regression models with dispersion models; results are inconsistent		
	·Residential mobility, time-activity pattern, and exposure at non-residential addresses should be evaluated in exposure assessment		
	·Assessment of long-term validity (i.e. stability) of land-use regression models which are based on one measurement campaign		
	·There is currently little validation of modeled exposures against personal exposure measurements		
Water contamination	·Best method for exposure assessment is to combine information of water-related behaviors obtained by questionnaire with newly or routinely collected water contaminant measurements	·Further opportunity to study exposure to water pollutants in cohorts without water exposure assessment; routinely collected water pollutants are often available	·Assessment of exposure to substances such as pharmaceuticals, Perfluorooctanesulfonic acid (PFOS)/Perfluorooctanoic Acid (PFOA), and (other) endocrine disrupters
	·Validation of questionnaires against biomonitoring since it has been conducted in only a few subjects	·Consideration of source of water as an exposure indicator (ground water versus surface water)	
	·Assessment of variability and measurement error of questionnaires by repeated assessments	·Data pooling currently being done in the HIWATE project	
		·Access to publicly collected data should be increased and European databases should be made available to researchers	
Allergens & microbial agents	·Measurements in house dust or air samples are recommended	·Meta-analyses in the framework of the GA2LEN (allergens) and AIRALLERG projects (allergens and biocontaminants) and ENRIECO case study on mold and dampness	·Use of newly developed analysis techniques such as molecular methods or DNA fingerprinting
	·Use of questionnaires is inexpensive, but questionnaires were found to have a low sensitivity		
	·Exposure at non-residential locations and timing of exposure should be taken into account		
Metals	·Human biological monitoring is the state of the art method for estimation of total dose.	·Data pooling and/or meta-analysis of the data available in the European birth cohorts can overcome this problem if conversion models can be developed to transfer between different biological media (hair, cord blood, urine, etc.).	·Validation of questionnaire data against human biological monitoring is needed.
	·Inductively coupled plasma mass spectrometry (ICP-MS) is more sensitive and faster than Atomic absorption spectroscopy (AAS)		
	·In general labs are using well standardized protocols. It is recommended to validate the analytical technique in each lab including a sample with known concentration of metal/s every X number of samples. This will be useful to validate both the pre-treatment and the analytic process.		
	·Some studies compared Mercury levels in biological samples with fish/shellfish consumption assessed through validated food frequency questionnaires with encouraging results. More studies are needed to confirm these findings. For other metals, validation of other exposure assessment methods must be further explored.		
Pesticides	·There are multiple pesticides and multiple pathways of exposure conducing to varying exposure assessment	·Since few cohorts assessed exposure to pesticides there is a large scope of doing more work on pesticide exposure within the European birth cohorts, particularly by analyzing available biological samples	·Include validation studies in exposure assessment
	·Biomonitoring hardly feasible for large cohorts, but recommended on sub-populations for validation purposes and identification of main exposure sources	·Use of Geographic Information System (GIS) technologies with existing European data on soil occupancy and satellite imagings/maps of crops to assess bystander exposure due to agricultural activities	·Assessment of time-activity pattern and exposure at non-residential locations
	·There is a need for harmonization of exposure assessment by biomonitoring (choice of molecules of interest, biological matrices, sampling and storage conditions, chemical analyses controlled by international comparison programs, etc.) between studies		
	·Validation of questionnaires needed		
	·Inclusion of exposure at non-residential locations may improve exposure assessment		
Persistent organic pollutants	·High-performance liquid chromatography (HPLC) derived methods are the state of the art for measurements of POPs	·The possibility to perform a pooled or meta-analysis on the association between exposure to POPs and birth outcomes in the European birth cohorts have been evaluated within a case study that is part of ENRIECO and continued in CHICOS (http://www.chicosproject.eu).	·There is high degree of variability between studies in study design including timing of sample collection and collection medium. Therefore additional data collection according to a standardized protocol may be needed, especially if the outcomes of interest are hypothesized to be related to exposure at specific time windows during fetal life or early childhood.
	·All analyzing laboratories should participate in inter-laboratory calibration tests.	·Data pooling is possible for polychlorinated biphenyl (PCB), and dichlorodiphenyltrichloroethane (DDT). For other POPs there is little data or too much heterogeneity with regard to the sampling media or the timing of exposure assessment.	
	·Especially for detecting POPs in low concentrations in small volumes equipment with a high sensitivity is needed.	·Conversion factors needed to be developed to allow pooling of data.	
	·The persistence of organochlorines makes sample degradation a lesser problem as for other more readily degradable compounds. However, it is recommended to store samples at −80°C at least, if measurements are planned to be performed after several years.		
Other chemical exposures (brominated flame retardants, perfluorinated compounds, phthalates and phenols)	Human biological monitoring is the state of the art method for estimation of total dose	There is currently little published data in the cohorts, but many measurements are ongoing and we recommend cohorts starting to work towards combined and comparison studies.	This is an emerging field and there is a rapidly growing expertise in the cohorts, which would benefit from continued communication and coordination.
	·For non-persistent exposures with very short half-lives (phthalates and phenols), we recommend repeated measurements as standard practice.	·Conversion factors should be developed to transfer from concentrations in one medium/time point to another in order to compare/combine data from different cohorts.	·Very little is known about the effects of postnatal exposure to emerging chemicals, and therefore we recommend further evaluation of these new chemicals in children.
	·Issues of contamination from storage materials and lab equipment, and storage conditions are of great importance and need to be addressed in depth. We recommend to closely follow published recommendations on sampling collection and packing, storage, and analysis (see COPHES website: http://www. eu-hbm.info/cophes).		·Active dialogue and partnership among the scientists representing the various disciplines would be essential for selecting new contaminants and setting prioritization for measurement in birth cohort studies.
	·It is recommended to conduct a European evaluation of inter- and intra-laboratory variability.		
	·Validation of other exposure assessment methods such as questionnaires, occupational Job Exposure Matrices (JEMs), environmental measurements, and/or toxicokinetic models is needed		
Radiation	*Ionizing radiation*		
	·Assessment of medical radiation exposures (X-ray, computer tomography (CT)-scan) by standardized questionnaires	·Existing data are not sufficient for pooled studies.	·Assessment of medical radiation exposure
	·Assessment of occupational exposure by means of badge dose information or if not possible by questions on x-ray equipment and protective equipment used		·Evaluation of link with other EUROPEAN cohorts of children exposed to CT scans
	·Assessment through job-exposure matrices difficult		
	*UV*		
	·ENRIECO has developed a set of core questions on sun exposure for different exposure-time windows that is recommended for use in cohort studies	·Existing data are not sufficient for pooled studies	·Inclusion of vitamin D and UV exposure related questions in cohort questionnaires.
	*Non-ionizing radiation*		
	·Use of core set of standardized questions to assess mobile and cordless phone use	·Comparison studies with existing data comparing questionnaire data between cohorts	·Integration of standardized questions on use of mobile phones to facilitate future combined analyses of non-cancer effects
	·Validation of questionnaires using information of other types of studies		·Collaborative efforts focusing on design of questions related to other RF-EMF sources (e.g. WiFi, new communication technologies, microwave ovens, baby phones)
	·Coordination between cohorts in developing validated exposure models for RF and ELF-EMF		
Smoking and second hand tobacco smoke	·Questionnaires are most suitable method for larger epidemiological studies and for assessment of long-term exposure	·Combined analyses on the effects of pre- and postnatal exposure to second hand tobacco smoke have been performed within a case study that is part of ENRIECO	·Large studies with close monitoring of second-hand tobacco smoke exposure before conception, during trimesters of pregnancy and during the first year of life to disentangle the role of exposure during different periods
	·Relevance of the timing of the exposure (before conception; during pregnancy, infancy, childhood or later in life) is not clear. Large studies can enhance knowledge if exposure is assessed repeatedly during different time periods.		·Specific questions recommended for the different exposure periods
Noise	·Objective measures should be in accordance with the European Union’s Environmental Noise Directive (END) guidelines.	·Few European cohorts currently have data from objective noise assessments that could be combined	·Inclusion of objective and subjective exposure assessments
	·Noise propagation modeling is recommended for large studies.		·Assessment of time-activity pattern
	·Questionnaire- assessments of noise annoyance should be performed in addition to objective measures; standard scales can be recommended. Information on non-residential exposure, time-activity pattern, and insulation of buildings, window opening behavior and the position of bedroom in relation to source of noise should be included in exposure assessment.		
Occupational exposures	·A number of Job Exposure Matrices (JEMs) have been built in Europe covering different periods of time and different types of exposures.	·Within the ENRIECO project, the possibility to perform pooled/meta-analyses of the association between adverse health outcomes and selected occupations of mothers and fathers during vulnerable periods has been explored; 14 cohorts are eligible for this analysis, 12 have already expressed their interest. A protocol for the analysis has been developed.	·For an adequate data collection on occupational exposures job title is not sufficient. In addition, one should collect description of task, type of industry, number of hours per week, and if possible name of company, existence of biomonitoring data. Free text should be kept in the data base for additional details.
	·JEMs need to be validated against objective measures of exposure (work environment, biomarkers)		·A good training of coders should be organized for harmonization of occupation coding
	·If JEMs are used, they should be country- and agent-specific since work environments differ between countries and time periods.		·Standardized questionnaires for physical load should be published
	·To avoid any influence of birth outcome on the availability of occupational information and on its quality, we recommend that data should be collected before birth. This is mandatory when questionnaires on occupational exposures are used and optional for job title.		
	·The period of interest is around conception and each trimester - or at least one trimester of pregnancy (depending on the health outcome studied) for mothers, and before conception for fathers.		

## Discussion

The inventory revealed that in the European birth cohort studies, rich and diverse data on environmental exposures exist. Several examples of successful collaborations were identified making use of exposure data obtained in several cohorts.

A number of methodological issues were identified as well. Often, indirect methods were used rather than measures of personal exposure. Temporal misalignment of exposure measurements relative to timing of health measurements was also identified, as was lack of information on time-activity patterns. All of these produce error in exposure measurements which may attenuate risk estimates and statistical power of a study and increases the likelihood that real associations are not detected [2]. The National Research Council ranked the different direct and indirect approaches of exposure assessment and considered personal exposure measurements as the best estimate of actual exposure [29]. Exposure assessment by means of (repeated) individual environmental or biological measurements, however, is costly and therefore usually not feasible in all participants of large cohort studies. Therefore, in many studies in this inventory, questionnaires were used to enquire about the exposure of interest, occasionally in combination with environmental measurements (water contamination).

Questionnaire reports were found to be an inexpensive and valid estimate of residential SHS exposure among preschool and school children [9-11], whereas questionnaire-reported cat and dog ownership is a relatively poor measure of pet allergen levels in house dust [13-16] and cannot be used as a surrogate for measurements of specific microbial agents in house dust [17-19]. House dust collection by study participants instead of fieldworkers can reduce the costs associated with the collection of dust samples for the assessment of allergen and biocontaminant exposure. Several methods have been described in the literature including nylon socks [30], electrostatic wipes [31,32], and passive samplers [33]. For other exposures such as household use of pesticides and non-ionizing radiation, where exposure assessment also largely relies on questionnaires, such validation studies are still lacking. Also, for chemical exposures such as phthalates that are currently exclusively measured by biomonitoring, no validated questionnaires exist and predictors need to be identified [7].

Likewise, there is still little validation of modeled exposures (ambient air pollution) or surrogate variables (e.g. proximity to agricultural activities as a proxy for bystander exposure to pesticides) against individual environmental monitoring.

As the birth cohorts studies in this inventory were all funded locally, there was no initial harmonization of exposure assessment methods For example, different questionnaires were used in different cohorts; there were no standardized protocols for the collection and analysis of individual environmental samples and biomonitoring was done in different media such as breast milk, cord blood, placenta, serum and whole blood. Some exceptions include studies in which a standardized exposure assessment was part of a collaborative effort (e.g. TRAPCA (outdoor air pollution [21]), ESCAPE (outdoor air pollution [22,23,34]), HIWATE (water contamination [24]) AIRALLERG (allergens, biological contaminants and indoor air pollution [25,26]), and HITEA (indoor biological agents, http://www.hitea.eu/). In the absence of such prior harmonization of methods, data can still be combined after careful examination of the communalities and differences between methods. Moreover, it should be noted that harmonization of exposure assessment is not straightforward and may not be beneficial in all cases as for example for many exposures there is no gold standard, and questionnaires may be based on wrong hypotheses. This is more a concern for emerging exposures (e.g. radiation) than for established exposures (e.g. SHS). Furthermore, the development of internationally accepted standards is a complicated and lengthy process and often standards hinder the development and introduction of new methods. Especially for the assessment of emerging exposures and for new exposure assessment methods diversity is desired as it allows the evaluation and comparison of different methods. For exposures that are (mainly) assessed by means of biomonitoring (i.e. metals, pesticides, POPs, and other chemical pollutants), the performance of inter-laboratory comparisons and either the harmonization of the exposure assessment with regard to the sampling medium or the development of conversion factors has been recommended to facilitate combined analyses (Table 2).

Individual assessment of exposures experienced by the study subjects in different micro-environments by means of personal or stationary monitoring alone will generally not be feasible in birth cohorts, as the study populations generally comprise several hundreds to thousands of subjects. Therefore, environmental exposure assessment in the European birth cohorts currently is often limited to residential exposure although many study participants regularly spend considerable amounts of their time outside their homes for instance at day care centers or schools. Consequently, little is known about the role of non-residential exposure and time-activity pattern in the association between these exposures and health. Some recent publications on the effects of ambient air pollution where exposure was estimated as a time-weighted average of several addresses where the participants spent considerable amounts of time indicated little differences between the estimated exposure at the home address only and the time-weighted average of residential and non-residential (i.e. work or school) exposure [35-37]. However, this needs further evaluation.

For many exposures, we presently know very little about the relevance of the timing of the exposure in addition to the level of exposure, and it is unclear whether exposure during a specific period when organs develop and are considered being more susceptible, is more important than later exposure (e.g. for congenital anomalies early pregnancy and birth weight mostly likely late pregnancy). The window of susceptibility for reproductive outcomes is most likely short (days, months, trimesters of pregnancy) and depends on the type of outcome [38]. For asthma and allergies it has been hypothesized that there is a “window of opportunity” early in life where the development of asthma and allergies is initiated by a variety of factors [39]. However, we cannot rule out that there are multiple windows of susceptibility during fetal development and (early) childhood. As an example, one of the case studies performed within the ENRIECO project demonstrated that SHS exposure during pregnancy and during the first year of life are independent risk factors for childhood wheeze and asthma [28].

Prospective birth cohort studies with repeated exposure and health outcome assessments offer a unique possibility to increase our knowledge with regard to the temporal variability of exposure and if variability is sufficient the relevance of exposure during different time periods. The need for repeated exposure assessments depends on the temporal variability and the toxicokinetics of the exposure of interest: if there is little variability, few repeated measures are needed; if there is a lot of variability many repeated measurements are needed. The number of measurements that can be performed in a cohort study, however, is limited, and therefore it will never be possible to continuously monitor exposure. Repeated exposure assessments, for part of the population if not possible for all study participants, however, can provide valuable information about the validity of a single exposure assessment for a longer period, i.e. its ability to predict concentrations in earlier of later periods. For example, land-use regression models that are currently used very often for assessments of long-term exposures to outdoor air pollution are based on one measurement campaign during which air pollution concentrations are measured at a number of locations. Few validation studies have been performed so far. Recently, it has been shown that land-use regression models were highly predictive of NO/NO_2_ concentrations measured 10 and 13 years apart in The Netherlands, and Rome, Italy [40,41]. A high correlation has also been shown between measurements of POPs performed as much as 10 years apart [42]. Furthermore, there is some evidence that a single endotoxin [43-46], mite or cat allergen measurement [46,47] is a valid estimate of exposure for longer time periods.

Collecting data before the occurrence of any adverse health event of interest is crucial when temporal variability of the exposure of interest is high or the presence of a certain disease or condition can result in changes in exposure (e.g. allergen avoidance in subjects with asthma and allergies). Furthermore, if exposure assessment relies on (parental) self-reporting a retrospective assessment may result in reporting bias or recall bias (e.g. due to increased awareness of certain exposures in diseased subjects). Nevertheless, also in prospective cohort studies, exposure assessment is sometimes done retrospectively, i.e. an exposure assessment is added to existing health data because time and money for exposure assessment at the beginning of a study are limited and often new exposures become of interest only after the study has been going on for some time. The storage of (part of) biological and environmental samples for later analyses as well as the use of historical data that has been routinely collected for other (e.g. monitoring) purposes can overcome the problems associated with retrospective exposure assessment. Another possibility included the use of GIS-based techniques and exposure modeling techniques, which are currently in particular, but not exclusively, used for air pollution exposure assessment. For example, within European birth cohort studies, GIS-based techniques have also being used for assessment of noise. Furthermore, outside European birth cohorts, GIS-based techniques have been used to assess pesticide exposures, e.g. [48,49] and more recently exposure to radiofrequency electromagnetic fields [50].

Lessons from collaborative efforts so far have been that combining data from various cohorts requires careful consideration of the aims, protocols, data (comparability and availability of exposure, health and relevant confounder data), ethical issues, analyses and management, and it is time and labor intensive but potentially fruitful. As an example, a challenge of the case study on POPs [6] was the development of conversion factors to facilitate combined analyses with persistent organic pollutant measurements performed in different media. Collaborative studies performed within the EU-funded CHICOS project (http://www.chicosproject.eu) currently build on these experiences. Both, existing collaborative studies as well as our recommendations regarding future meta-analyses and/or pooled analyses within the European birth cohort studies, so far, were very much focused on the study of one exposure at a time. Possible interactions between different exposures are of course of major interest as environmental exposure is not limited to a single agent and basically all cohorts have data on multiple exposures. Limited statistical power of single cohort studies is a much bigger issue in the study of interactions between exposures than in one-agent-at-a-time studies, resulting in an even greater need for collaborative studies here.

Substantive questions in environmental health that could potentially be answered by future collaborative efforts include health effects of ultrafine particles in air; pharmaceuticals, PFOS/PFOA and other endocrine disruptors in drinking water [51]; and medical radiation exposure.

Improvement is needed of questionnaire instruments to assess water contamination, UV, non-ionizing radiation, second hand tobacco smoke, noise, and occupational exposures. Inter-laboratory comparisons are needed for methods to measure POPs and other chemicals through biomonitoring. Recommendations for future work include the use of new technologies such as GIS and satellite imaging for assessment of pesticide exposure and molecular methods or DNA fingerprinting for assessment of microbial exposures.

Lack of information on variables that are determinants of exposure *and* health outcomes can lead to confounding bias in epidemiological studies [8]. A discussion of relevant confounders for a wide range of exposure-health relationships is beyond the scope of this paper. However, there are two variables that may act as a confounders of many exposure health relationships and that we would like to mention here, namely socio-economic status (SES) and genetic predisposition. Socio-economic status has been shown to be an important determinant of several environmental exposures (e.g. air pollution and second-hand smoking) and susceptibilities (e.g. pre-existing health conditions, stress, behavior), which have been suggested to act together to influence the health response of groups classified by socioeconomic level [52-54]. Therefore information on SES has been collected in basically all existing cohorts. We strongly recommend to new cohorts to collect individual information on participants’ SES (e.g. parental education, income or occupation) to assess potential confounding and modifications of exposure-health relationships by SES. Likewise, information on genetic predisposition is very important and should be collected as genetic predisposition may act as a confounder (e.g. allergen-avoidance of allergic parents [55]) or an effect modifier of the association of interest.

## Concluding remarks

European birth cohorts are collecting a wealth of data on environmental exposures. Most data is currently available for outdoor air pollution, allergens and microbial agents, smoking and second hand tobacco-smoke, and occupational exposures. Collaborative analyses with data from several birth cohorts have been performed successfully for outdoor air pollution, water contamination, allergens, biological contaminants, molds, POPs and tobacco smoke exposure. This illustrates the large potential for collaborative analyses of other environmental health issues as well. Investigators can use this review to evaluate the potential for future collaborative analyses with respect to data availability and methods used in the different cohorts and to identify potential partners for a specific research question. The main reasons for collaboration between population studies are replication, studying heterogeneity, and increasing statistical power to study small relative risks, rare events and/or complex interactions. Apart from subject-matter specific recommendations, progress can only be achieved with further harmonization of methods, including those for environmental exposure assessment. The ENRIECO project shows the potential as well as the limitations to use data from existing, locally funded studies for this. Assuming that the majority of future studies will continue to be locally designed and funded, there is a need to periodically review methods for exposure assessment as they become available. Investigators and funding agencies can then make use of this information to choose methods.

## Abbreviations

AAS: Atomic absorption spectroscopy; CT: Computer tomography; DDT: Dichlorodiphenyltrichloroethane; END: Environmental Noise Directive; ELF: Extreme low frequency; EMF: Electromagnetic field; ENRIECO: Environmental Health Risks in European Birth Cohorts; GIS: Geographic Information System; HPLC: High-Performance Liquid Chromatography; ICP-MS: Inductively Coupled Plasma Mass Spectrometry; JEM: Job Exposure Matrix; PCB: Polychlorinated Biphenyl; PFOA: Perfluorooctanoic Acid; PFOS: Perfluorooctanesulfonic Acid; POP: Persistent Organic Pollutant; RF: Radio Frequency; SES: Socio-Economic Status; SHS: Second Hand Smoke; UV: Ultraviolet.

## Competing interests

The authors declare that they have no competing interests.

## Authors’ contributions

MN was the Principial Investigator of the ENRIECO project. MV and MC were responsible for the inventory that served as a basis for the evaluations presented in this paper. All authors were responsible for the evaluation of the information for one or more exposure topics including the writing of reports. UG wrote the manuscript; all other authors critically reviewed the manuscript and approved the final version of the manuscript for submission.

## Supplementary Material

Additional file 1 Environmental exposure assessment in European birth cohorts: results from the ENRIECO project - Additional Material Table of names of experts participating in reviews and additional tables describing environmental exposure assessment in European birth cohorts. Click here for file
