# Supplementary material for: Environmental exposure assessment in European birth cohorts: results from the ENRIECO project
Source: Environ Health. 2013 Jan 23;12:8. doi: 10.1186/1476-069X-12-8 (PMC3564791; doi:10.1186/1476-069X-12-8)
Supplement: Additional file 1 — Environmental exposure assessment in European birth cohorts: results from the ENRIECO project - Additional Material Table of names of experts participating in reviews and additional tables describing environmental exposure assessment in European birth cohorts. [file 1476-069X-12-8-S1.pdf]

Environmental exposure assessment in European birth cohorts: results from the ENRIECO project

Additional Material

Ulrike Gehring[1], Maribel Casas[2,3,4], Bert Brunekreef[1,5], Anna Bergström[6], Jens Peter Bonde[7], Jérémie Botton[2,3,4], Cecile Chevrier[8], Sylvaine Cordier[8], Joachim Heinrich[9], Cynthia Hohmann[10], Thomas Keil[10], Jordi Sunyer[2,3,4,11], Christina G. Tischer[9], Gunnar Toft[12], Magnus Wickman[6], Martine Vrijheid[2,3,4], Mark Nieuwenhuijsen[2,3,4]

Affiliations

1 Institute for Risk Assessment Sciences, Utrecht University, Utrecht, The Netherlands

2 Centre for Research in Environmental Epidemiology (CREAL), Barcelona, Spain

3 Hospital del Mar Research Institute (IMIM), Barcelona, Spain

4 Spanish Consortium for Research on Epidemiology and Public Health (CIBERESP), Spain

5 Julius Center for Health Sciences and Primary Care, University Medical Center Utrecht, The Netherlands

6 Institute of Environmental Medicine, Karolinska Institutet, Sweden

7 Department of Occupational and Environmental Medicine, Bispebjerg University Hospital, Copenhagen NV, Denmarkv

8 INSERM U1085 - IRSET, University of Rennes I, France

9 Helmholtz Zentrum, München & German Research Centre for Environmental Health, Institute of Epidemiology I, Neuherberg, Germany

10 Institute of Social Medicine, Epidemiology and Health Economics, Charité University Medical Center Berlin, Germany

11 Universitat Pompeu Fabra (UPF), Spain

12 Department of Occupational Medicine, Aarhus University Hospital, Denmark

Table S1. Experts who participated in the different working groups.

Exposure	WG-group leader	Participants[a]
Outdoor air pollution	Ulrike Gehring<br>u.gehring@uu.nl	Ferran Ballester, Bert Brunekreef , Ann Colles, Vicky Patelarou, Frank Pierik, Kinga Polanska
Water contamination	Mark Nieuwenhuijsen<br>mnieuwenhuijsen@creal.cat	Jérémie Botton, Maribel Casas, Sylvaine Cordier, Vicky Patelarou,
Allergens & microbial agents	Joachim Heinrich<br>joachim.heinrich@helmholtz-muenchen.de	Chih-Mei Chen, Ulrike Gehring, Christina Tischer, Constantine Vardavas
Metals	Jordi Sunyer<br>jsunyer@creal.cat	Ferran Ballester, Maribel Casas, Kinga Polanska, Constantine Vardavas, Michael Wilhelm
Pesticides	Cecile Chevrier<br>cecile.chevrier@rennes.inserm.fr	Sylvaine Cordier, , Claire Petit, Frank Pierik, Stephanie Vandentorren
Persistent organic pollutants	Gunnar Toft<br>gunntoft@rm.dk	Jens Peter Bonde, Merete Eggersbø, Hannu Kiviranta, Vicky Patelarou, Greet Schoeters, Remy Slama, Stephanie Vandentorren, Michael Wilhelm
Other chemical exposures	Martine Vrijheid<br>mvrijheid@creal.cat	Maribel Casas, Elly Den Hond, Ilias Kavouras, Frank Pierik, Stéphanie Vandentorren, Gunnar Vase Toft, Michael Wilhelm
Radiations	Martine Vrijheid<br>mvrijheid@creal.cat	Elisabeth Cardis, Maribel Casas, Payam Dadvand, Mark Nieuwenhuijsen<br>External experts: Anke Huss, Roel Vermeulen
Smoking and second hand tobacco smoke	Magnus Wickman<br>magnus.wickman@ki.se	Anna Bergström, Ulrike Gehring. Eva Hallner, Cynthia Hohmann, Thomas Keil, Åsa Neuman, Mattias Öberg, Göran Pershagen
Noise	Thomas Keil<br>thomas.keil@charite.de	Maria Pia Fantini, Cynthia Hohmann, Yvonne de Kluizenaar, Lorenza Luciano, Frank Pierik
Occupational exposures	Sylvaine Cordier<br>sylvaine.cordier@univ-rennes1.fr	Jens Peter Bonde, Lex Burdorf, Merete Eggesbø, Ana Maria Garcia, Regina Gražulevičienė, Manolis Kogevinas, Mark Nieuwenhuijsen, Anne-Marie Nybo-Andersen, Vicky Patelarou, Frank Pierik, Ana Cristina Santos, Claudia Snijder, Martine Vrijheid, Tanja Vrijkotte<br>External experts : Joëlle Févotte, Hans Kromhout, Nel Roeleveld, Martie Van Tongeren

[a] in alphabetic order

Table S2. Modelling of outdoor air pollution in European birth cohorts.

Cohort	NO_2	NO_x/NO	$PM_{2.5}$	PM_{coarse}	PM_{10}	"Soot"	SSE	Others	N [a,b]	Timing [b]
ABCD	●◊	◊	◊	◊	◊	◊	◊		~ 7,600	Pregnancy & birth
BAMSE	●◊	◊▲	●◊	◊	◊▲	●◊	◊	▲ SO_2	~3,700	1yr, 4-8 yrs
BiB	◊	◊								
DNBC	◊▲	◊▲	◊	◊	◊	◊	◊	▲ Ozone	~18,000	0-1.5 yrs
EDEN	●◊▲	◊			▲			∆ Benzene	700-1,000	Pregnancy
ELFE	∆	∆	∆		∆			∆ Ozone		
GAPS II	●▲	●▲	▲		▲	▲		▲ Ozone	581-708	Birth-4 yrs
Generation R	◊▲	◊	◊	◊	◊▲	◊∆	◊		~9,000	All ages
GINIplus	●◊	◊	●◊∆	◊	◊	●◊	◊		3,878-5,991	Birth, 1, 2, 4, 6 yrs
INMA old										
Granada	●◊	●◊							220	4 yrs
Menorca	○	○								
INMA new										
Asturias	●◊	◊	○		○			● Benzene; ○ TEX	478	Pregnancy
Gipuzkoa	●◊	◊						● Benzene	597	Pregnancy
Sabadell	●◊	●◊						● BTEX	608	Pregnancy & any age
Valencia	●◊	◊	◊	◊	◊	◊	◊	○ BTEX	785	Pregnancy
KANC	◊	◊▲	◊▲	◊	◊▲	◊	◊		4,000	Pregnancy & birth
Leicester	○	○			○▲				~1,000	1and 4 yrs
LISAplus	● ◊	◊	●◊	◊	◊	●◊	◊		1,314-2,357	0, 2, 6 yrs
MOBA	◊	◊	◊	◊	◊	◊	◊			
NINFEA	◊	◊	◊	◊	◊	◊	◊			
PARIS	◊	▲	◊		◊					All ages
PIAMA	●◊	◊	●◊	◊	◊	●◊	◊		3,953	Pregnancy & birth
RHEA	◊	◊	◊	◊	◊	◊	◊			Pregnancy & birth

[a] number of children with exposure information

[b] only for work that has been done already

● Land-use regression (LUR) done (outside ESCAPE); ○ LUR planned (outside ESCAPE); ◊ LUR ongoing work within ESCAPE; ▲Dispersion modelling done; ∆ Dispersion modelling planned

PM_{coarse} = PM_{10}-$PM_{2.5}$; Soot = reflectence of $PM_{2.5}$ filters or elemental carbon; (B)TEX = (benzene,) toluene, ethylbenzene, xylenes; SO_2 = Sulfur dioxide; SSE = "source specific" elements from X-ray fluourescence

mo = months, yrs = yrs

Table S3. Assessment of exposure to allergens and microbial agents by means of environmental monitoring in European birth cohorts.

	Component measured (time point[a])						
	Cat	Dog	House dust mite	Mould	Bacteria	Other	N (time point)[b]
BAMSE	Fel d1 (2 mo, 7 yrs)	Can f1 (2 mo)	Der p1 & Der f1 (2 mo, 7 yrs)	β(1,3) glucan, EPS *Asp/Pen* (7 yrs)	Endotoxin (7 yrs)		533 (2 mo), 358 (7 yrs)
ELFE				Alternaria, Aspergillus, Cladosporium, Penicillium, Stachybotrys chartarum			0-1 yr *
GINIplus	Fel d1		Der p1 & Der f1	β(1,3) glucan, EPS *Asp/Pen*	Endotoxin		219 (5-6 yrs)
INMA old							
Menorca	Fel d1		Der p1				482 (3 mo), 11 yrs*
INMA new							
Asturias	Fel d1		Der p1			Pollen	4 yrs*
Sabadell	Fel d1		Der p1			Pollen	14 and 30 mo.*
Krákow	Fel d1	Can f1	Der p1 & Der f1				336 (3 yr), 250 (5 yr)
LISAplus	Fel d1 (3 mo, 5-6 & 10 yrs)		Der p1 & Der f1 (3 mo, 5-6 yrs)	β(1,3) glucan, EPS *Asp/Pen* (5-6 & 10 yrs)	Endotoxin (3 mo, 5-6 & 10 yrs)		2,166 (3 mo), 138 (5-6 yrs), 100 (10 yrs)
LUKAS	Fel d1		Der p1 & Der f1	EPS *Asp/Pen*	Endotoxin		~350 (2 mo, 1yr, 3 yrs, 6 yrs)
MAS	Fel d1	Can f1	Der p1				1,000 (3mo), 800 (18 mo), 600 (3 yrs)
PARIS			Der p1 & Der f1	Alternaria, Aspergillus, Cladosporium, Penicillium	Endotoxin	Pollen	162-196 (1 mo), 157-196 (6 or 9 mo)
PIAMA	Fel d1 (pregnancy, 3 mo, 1, 4, 5-6 & 8 yrs)	Can f1 (pregnancy, 3 mo, 1, 4, 5-6 & 8 yrs)	Der p1 & Der f1 (pregnancy, 3 mo, 1, 4, 5-6 & 8 yrs)	β(1,3) glucan, EPS *Asp/Pen* (pregnancy, 3 mo, 1 & 5-6 yrs)	Endotoxin (pregnancy, 3 mo, 1 & 5-6 yrs)		Allergens/mould,bacteria: 700/200 (pregnancy), 1,750/700 (3 mo.), 200/100 (1 yr), 1,000 (4 yrs), 350 (5-6 yrs), 100 (8 yrs)

[*] planned/ongoing

[a] if components were measured at different time points

[b] N = number of children with exposure information; only for work that has been done already; excluding ongoing/planned work

Can f = Canis familiaris; Der p = Dermatophagoides pteronyssinus; Der f = Dermatophagoides farinae; Fel d = Felis domesticus;

EPS *Asp/Pen* = Extracellular polysaccharides from *Aspergillus* and *Penicillium spp*.

mo = months, yrs = yrs

Table S4. Biological and environmental monitoring for assessment of exposure to smoking and second hand tobacco smoke in European birth cohorts.

Cohort	Component	Medium	Timing [a] (N [a,b])
**Biological monitoring**			
ALSPAC	Cotinine	B	7 yrs (7,000, child), 15 yrs (3,500, child)
BAMSE	Cotinine	U	4 yrs (990, child)
Czech	Cotinine	B	Birth (293, mother)
		U	3-5 yrs (523, child), 6 yrs (980, child), 10 yrs (940, child)
Duisburg	Cotinine *	U	
	Nicotine *	U	
EDEN	Cotinine *	U	
ELFE	Cotinine *	U	
GINIplus	Cotinine	U	5-6 yrs (199, child)
INMA old			
Granada	Cotinine	U	4-5 yrs (174, child)
Ribera Ebre	Cotinine *	U	
INMA new			
Asturias	Cotinine *	U	
Gipuzkoa	Cotinine *	U	
Sabadell	Cotinine *	U	
Valencia	Cotinine *	U	
INUENDO	Cotinine *	B	
Krákow	Cotinine	B	Pregnancy (467, mother)
		C	Birth (484)
		U	3 yrs (219, child)
LISAplus	Cotinine	U	5-6 yrs (119, child)
REPRO_PL	Cotinine	S	Pregnancy (1,800, mother)
RHEA	Cotinine	H	Pregnancy (500-600, mother)
		U	6-24 mo.(300, child)
	NNAL	U	Pregnancy (150, mother)
**Environmental monitoring**			
BAMSE	Nicotine	A	5-9 yrs (356)
GINIplus	Nicotine	A	5-6 yrs (215)
LISA	Nicotine	A	5-6 yrs (137)
PARIS	Nicotine	A	1-6 mo (196), 9-12 mo (196)
PIAMA	Nicotine	A	1-2 yrs (90), 5-6 yrs (341)

* planned;

[a] only for work that has been done already

[b] number of children with exposure information, mother/child indicates that sample was taken from the mother /the child

A=air; B = blood/serum; C = cord blood; H = hair; S = saliva, U = urine

NNAL = 4-methylnitrosoamino)-1-(3-pyridyl)-1-butanol

mo = months, yrs = years

Table S5. Assessment of exposure to water contaminants in Europan birth cohorts.

Cohort	DBPs	Metals	Nitrates	Pesticides	Endocrine disruptors	Others	Timing[a]
BiB	B,E,Q						Pregnancy
Duisburg	Q*						8 yrs
EDEN	E*,Q*						
ELFE	E*,Q*						
HUMIS						E (not determined)	
INMA old							
Granada	B,E,Q	E			E		Pregnancy, 4-5 yrs
Ribera Ebre				E			
INMA new							
Asturias	E,Q					E(Chlorine, physical parameters)	Pregnancy, birth, 1 yr, 4yrs
Gipuzkoa	E,Q	E,Q	E				Pregnancy, birth, 1yr, 4 yrs
Sabadell	E,Q	E	E				Pregnancy, 1yr, 4 yrs
Valencia	E,Q						Pregnancy, 1 yr., 4 yrs
KANC	E,Q	E,Q					Pregnancy
KOALA	Q						3 mo., 1 yr, 2 yrs, 5 yrs
MoBa	Q						Pregnancy
PARIS						Q* (Swimming pool water)	
PÉLAGIE	B,E,Q		E	B,E			Pregnancy
RHEA	E,Q						Pregnancy

[a] only for work that has been done already

* planned

DBP = Disinfection-by-products
B = biomonitoring ; E = environmental monitoring (routine monitoring companies or newly collected water concentration); Q = questionnaire

Table S6. Percentage [a,b] of cohort participants with biomonitoring data for water contaminants, metals, persistent organic pollutants (POPs), and other chemicals.

	Exposure				
**Cohort**	**No. of children at birth**	**Water contamin.**	**Metals**	**POPs**	**Other chemicals [d]**
ABCD	7,863				
ALSPAC	14,062		20-33%		3%
ArcRisk-Norway	430		66%	12-100%	
BIB	13,000	<1%			≤1%
DNBC	96,986			≤2%	
Duisburg	234		50-94%	35-96%	36-48%
EDEN	1,899		16-48%		10%
Faroes					
Cohort I	1,022		83-100%	44-100%	
Cohort II	182		90-100%	91-96%	
Cohort III	656		80-100%	76-100%	88%
FLEHS	1,196		100%	100%	
Generation R	9,778				1%
HUMIS	2,500			1-19%	15-16%
INMA old					
Granada	668	33%	15-17%	46-48%	4-7%
Menorca	482		62%	19-99%	19-51%
Ribera Ebre	102		70%	56-71%	
INMA new					
Asturias	485		71-99%	5%	6-11%
Gipuzkoa	611		83-100%	18-100%	5-18%
Sabadell	622		78-82%	4-100%	5-79%
Valencia	787		3-70%	22-69%	4-22%
INUENDO	1,322		30%	38-76%	27-38%
Kraków	505		48-91%	90-97%	
LUKAS	442		29%	17-29%	29%
MoBa	107,000				<1%
PCB cohort	1,134		9%	22-98%	18%
PELAGIE	3,421	18%	15%	17%	3-17%
REPRO_PL	1,800		17-78%	11%	
RHEA	1,500		3%		

[a] based on number of children at birth; [b] not including work that is planned; [c] brominated flame retardants, perfluorinated compounds, phthalates and phenols
